# Radiomics of Hepatocellular Carcinoma: Identifying Predictors of Microvascular Invasion Using Multi-Phase CT Analysis

**DOI:** 10.3390/jpm15110527

**Published:** 2025-11-02

**Authors:** Flavio Spoto, Nicolo’ Cardobi, Riccardo De Robertis, Luca Geraci, Luisa Tomaiuolo, Eda Bardhi, Beatrice Mascarin, Claudio Luchini, Andrea Ruzzenente, Mirko D’Onofrio

**Affiliations:** 1 Department of Diagnostics and Public Health Radiology Institute, Policlinico ‘G. B. Rossi’, Integrated University Hospital of Verona, P.le L.A. Scuro n. 10, 37134 Verona, Italybeatrice.mascarin@studenti.univr.it (B.M.);; 2 Department of Radiology, Ospedale Ca’ Foncello Treviso, Piazzale Ospedale 1, 31100 Treviso, Italyluisa.tomaiuolo@aulss2.veneto.it (L.T.); 3 Department of Diagnostics and Public Health, Section of Pathology, Policlinico ‘G. B. Rossi’, University and Hospital Trust of Verona, P.le L.A. Scuro n. 10, 37134 Verona, Italy; 4 Department of Surgery, Dentistry, Gynecology and Pediatrics, Division of General and Hepato-Biliary Surgery, Policlinico ‘G. B. Rossi’, University of Verona, P. le L.A. Scuro n. 10, 37134 Verona, Italy; andrea.ruzzenente@univr.it

**Keywords:** hepatocellular carcinoma, radiomics, microvascular invasion, computed tomography, texture analysis

## Abstract

**Objective:** To explore radiomic texture features from multi-phase contrast-enhanced CT as potential predictors of microvascular invasion (MVI) in hepatocellular carcinoma (HCC). **Materials and Methods:** This exploratory single-center study retrospectively analyzed 49 patients (54 HCC lesions) who underwent liver resection between 2018–2022. Radiomic analysis extracted 642 features across arterial, venous, and delayed phases using original and 5 mm-expanded tumor margins. **Results:** The 20–50 mm lesion subgroup (*n* = 37) provided the most reliable results, with arterial phase texture homogeneity features achieving AUC 0.772. Features from lesions <20 mm (*n* = 14, 4 MVI+) showed clear evidence of overfitting and were excluded from primary analyses. Delayed phase features showed preliminary associations (AUC 0.8) in a small LR-3/4 subset (*n* = 20). **Limitations:** This hypothesis-generating study has significant limitations including small sample size, single-center design, and lack of correction for multiple comparisons. **Conclusions:** Multi-phase CT radiomic analysis shows potential for MVI prediction in intermediate-sized HCC lesions, though external validation in larger cohorts is essential before clinical application.

## 1. Introduction

Hepatocellular carcinoma (HCC) represents the most prevalent primary liver malignancy worldwide. According to recent epidemiological data from GLOBOCAN 2020, HCC accounts for approximately 906,000 new cases and 830,000 deaths annually, ranking as the sixth most common cancer and third leading cause of cancer-related mortality globally [1]. The incidence increases with age across all populations, peaking at 70 years, with a consistent male-to-female ratio of 2:1. The economic burden of HCC is substantial, with management costs varying significantly based on disease stage and treatment modality. Early-stage disease amenable to resection incurs lower costs compared to advanced stages requiring systemic therapy or palliative care. The challenge of accurate preoperative staging, particularly MVI assessment, directly impacts treatment planning and resource allocation. Surgical resection remains the gold standard treatment for HCC [2,3], though significant limitations exist. Morbidity correlates directly with resection extent and preoperative functional status as classified by Child-Pugh criteria [4,5,6]. For patients with significant comorbidities, alternative treatments include radiofrequency ablation or alcohol injection [7]. Transarterial chemoembolization (TACE) addresses multifocal disease by exploiting tumor-specific vascularization patterns [8]. Histopathological markers are crucial for prognostic assessment. High tumor grades and microvascular invasion (MVI) are associated with increased recurrence risk, lymphatic metastasis, and tumor invasiveness [9,10]. Among prognostic factors—including grade, differentiation, size, multiplicity, and vascular invasion—MVI emerges as a critical independent predictor of recurrence-free survival following resection or transplantation [10,11]. However, reliable MVI diagnosis through biopsy remains challenging due to sampling errors [12]. Biopsy limitations for MVI detection warrant detailed consideration. As noted in our cohort where histopathological analysis revealed MVI in 52% of resected specimens, preoperative biopsy samples represent less than 1% of tumor volume, creating substantial sampling error. The heterogeneous distribution of microvascular invasion within tumors means that negative biopsy results cannot exclude MVI presence. Furthermore, biopsy carries risks including tumor seeding along the needle tract, hemorrhage, and pneumothorax, making it unsuitable for routine MVI assessment [12]. Given these diagnostic challenges and the critical prognostic importance of MVI, there is an urgent need for non-invasive imaging biomarkers that can reliably predict MVI preoperatively. This need has driven interest in advanced imaging analysis techniques, particularly radiomics, which can extract quantitative features from medical images that are imperceptible to visual assessment. The evolution of radiomics in oncology has progressed from initial texture analysis studies to sophisticated machine learning applications. Early investigations focused on single-phase acquisitions, primarily arterial phase imaging. Recent advances incorporate multi-phase analysis, though delayed phase evaluation remains largely unexplored despite its routine acquisition in many centers. Radiomics transforms medical images into high-dimensional, quantitatively analyzable data [13,14]. Radiomic features related to intensity, shape, size, volume, and texture can provide insights into tumor phenotypes and microenvironments. Integration with clinical data enables correlation with patient outcomes, enhancing evidence-based decision support [13].

The objective of this study was to identify radiomic parameters from multi-phase contrast-enhanced CT scans that can predict MVI in HCC, with particular emphasis on exploring the previously uninvestigated potential of delayed phase imaging features.

## 2. Materials and Methods

### 2.1. Study Design and Population

This retrospective single-center study screened 89 consecutive patients with radiologically diagnosed HCC who underwent liver resection between January 2018 and December 2022. The study was conducted in accordance with the Declaration of Helsinki and approved by the Ethics Committee for Clinical Experimentation of the Provinces of Verona and Rovigo (CESC) (approval number 4183CESC, protocol HepatobiliaryM&M).

Inclusion criteria comprised: availability of preoperative contrast-enhanced CT in DICOM format containing arterial, venous, and delayed phases; histopathological confirmation of HCC; and complete clinical data availability.

Exclusion criteria included: missing DICOM data or contrast phases; presence of biliary or duodenal metallic stents; and incomplete laboratory parameters.

### 2.2. Clinical Data Collection

Preoperative laboratory parameters included: alpha-fetoprotein (AFP), aspartate aminotransferase (AST), alanine aminotransferase (ALT), gamma-glutamyl transferase (GGT), total bilirubin, serum albumin, creatinine, and international normalized ratio (INR). Clinical parameters encompassed alcohol abuse history, hepatitis C virus (HCV) and hepatitis B virus (HBV) serology, ascites presence, cirrhosis status, hepatic encephalopathy, Model for End-Stage Liver Disease (MELD) score calculation, and Barcelona Clinic Liver Cancer (BCLC) staging.

Pathological data included capsule presence, differentiation grade, microvascular and macrovascular invasion status, resection margin status (R0: microscopically negative margins; R1: microscopically positive margins), and satellitosis. Radiological assessment documented maximum lesion diameter, capsular regularity, arterial enhancement pattern, and peritumoral washout according to LI-RADS criteria [15].

### 2.3. Imaging Protocol

All examinations were performed using a 64-slice CT scanner (Brilliance 64, Philips, Eindhoven, The Netherlands). Iodinated contrast medium (Ultravist 370, Bayer Schering Pharma, Berlin, Germany) was administered intravenously at 1.5 mL/kg body weight. Technical parameters included: 120 kV, 125–250 mAs, pitch = 1, and reconstruction thickness of 2 mm. Contrast timing employed a bolus tracking technique with acquisition at: arterial phase 15 s post-aortic threshold, portal venous phase at 60–70 s, and delayed phase at 180 s post-injection. Image acquisition parameters were standardized across all examinations: matrix size 512 × 512, field of view (FOV) 350–400 mm adjusted to patient size, rotation time 0.5 s, and collimation 64 × 0.625 mm. Image quality assessment criteria included: absence of motion artifacts, adequate hepatic parenchymal enhancement (>50 HU increase in portal phase), and complete liver coverage. Cases with suboptimal quality were excluded during initial screening.

### 2.4. Image Segmentation

**Figure 1:**
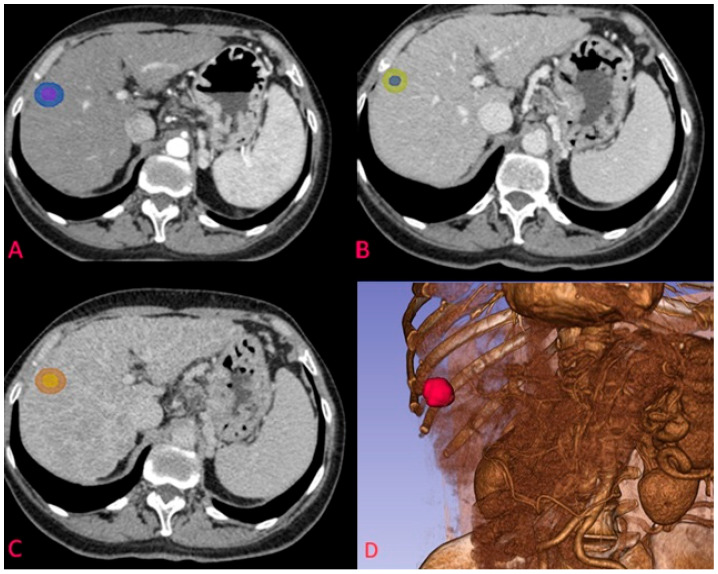
Example of segmentation of an HCC nodule in arterial phase at the S7-S8 junction. (**A**) nodule on transverse plane in arterial phase with corresponding 5 mm margin delineation; (**B**) segmentation of the same nodule in venous phase; (**C**) segmentation of the HCC nodule in delayed phase; (**D**) 3D reconstruction of the arterial phase and the segmented nodule.

A structured two-tier consensus protocol was implemented. Initial manual segmentation was performed by a radiology resident with <2 years of hepatic imaging experience using 3D Slicer software (version 5.3.0) across all contrast phases. Subsequently, two senior radiologists with >10 years of hepatic imaging experience independently reviewed all segmentations and jointly reached consensus on final volume delineations. While formal inter-observer variability metrics (e.g., Dice coefficient, intraclass correlation) were not calculated due to our consensus approach, we acknowledge this as a limitation. Future studies should incorporate systematic assessment of segmentation reproducibility. Regions of Interest (ROIs) were manually delineated slice-by-slice on axial images to create three-dimensional Volumes of Interest (VOIs), carefully excluding vessels, bile ducts, and extrahepatic regions. Dual segmentation was performed based on the finding that only 30% (16/54) of tumors had a defined capsule (Table 1). Comparative analysis justified this approach: for lesions 20–50 mm, features from 5 mm expanded margins (ART_5mm_GLCM_Idn, AUC = 0.772) outperformed those from original margins (ART_GLCM_Idn, AUC = 0.746) (Table 2), suggesting peritumoral tissue contains relevant information for MVI prediction. Figure 1 illustrates the segmentation process across all three contrast phases for a representative HCC nodule.

### 2.5. Radiomic Feature Extraction

Radiomic features were extracted using PyRadiomics (Computational Imaging and Bioinformatics Lab, Harvard Medical School, Boston, MA, USA). Features comprised eight categories: First Order Statistics (18 features), Shape-based 3D (14 features), Shape-based 2D (10 features), Gray Level Co-occurrence Matrix (GLCM, 22 features), Gray Level Run Length Matrix (GLRLM, 14 features), Gray Level Size Zone Matrix (GLSZM, 14 features), Neighboring Gray Tone Difference Matrix (NGTDM, 5 features), Gray Level Dependence Matrix (GLDM, 10 features). Each lesion yielded 107 features per phase, totaling 321 features for the three phases. Including 5 mm margin segmentations, the total feature count reached 642 per lesion. PyRadiomics extraction parameters included: bin width of 25 for discretization, minimum ROI size of 10 voxels, and no image resampling to preserve native resolution. Gray-level discretization used fixed bin width rather than fixed bin number to maintain reproducibility across different intensity ranges. Normalization was performed using z-score standardization within each ROI to account for inter-patient variability in contrast enhancement. Feature calculation employed 3D connectivity with 26-voxel neighborhood for texture matrices.

### 2.6. Statistical Analysis

Categorical variables were presented as frequencies and percentages. Continuous variables with normal distribution were reported as mean ± standard deviation (SD), while non-normally distributed variables were presented as median [interquartile range, IQR 25–75%]. The Shapiro-Wilk test assessed distribution normality. Student’s *t*-test was used to compare normally distributed continuous variables between MVI-positive and MVI-negative groups. The Kruskal-Wallis test addressed non-parametric comparisons. Due to the exploratory nature of this hypothesis-generating study, *p*-values are reported without correction for multiple comparisons. With 642 features analyzed per lesion, the expected number of false positives at α = 0.05 would be approximately 32 features by chance alone. To address this, a supplementary Bonferroni correction was performed (adjusted α = 0.05/642 = 0.000078) and no features remained significant after correction, confirming the preliminary nature of these findings. All results should therefore be interpreted as requiring independent validation with pre-specified hypotheses. Feature selection or dimensionality reduction (e.g., LASSO regression) was deliberately not performed in this exploratory phase to avoid potentially excluding features that might prove important in larger validation studies. This approach accepts a higher risk of Type I errors in exchange for comprehensive hypothesis generation. With 642 features and 54 lesions, the feature-to-sample ratio (approximately 12:1) far exceeds recommended ratios for predictive modeling. Receiver operating characteristic (ROC) curves evaluated significant parameters. Statistical analysis was performed using IBM SPSS Statistics v20.0. The significance threshold was set at *p* < 0.05.

## 3. Results

### 3.1. Patient Characteristics

Following application of inclusion and exclusion criteria, 49 patients (39 males, 10 females) comprised the final cohort, with a mean age of 68 ± 10 years. Five patients each contributed two lesions, totaling 54 HCC nodules for analysis. Cirrhosis was present in 71% (35/49) and HCV in 45% (22/49) of patients (Table 3). The median lesion diameter measured 25 mm [IQR 17.75–40], with liver segments S6 (21%) and S8 (17%) most frequently involved. According to the Barcelona Clinic Liver Cancer (BCLC) staging system, 26% of patients were classified as very early stage (BCLC 0), 59% as early stage (BCLC A), 13% as intermediate stage (BCLC B), and 2% as advanced stage (BCLC C). Histopathological characteristics of the lesions are detailed in Table 1.

### 3.2. Patient Stratification Analysis

Patients underwent dual stratification for analysis. By lesion size, we identified three categories: lesions <20 mm (14 lesions: 10 MVI-negative, 4 MVI-positive), lesions 20–50 mm (37 lesions: 16 MVI-negative, 21 MVI-positive), and lesions >50 mm (3 lesions: 0 MVI-negative, 3 MVI-positive). By LI-RADS classification, lesions were grouped as LR-3/4 (20 lesions: 11 MVI-negative, 9 MVI-positive) and LR-5 (34 lesions: 15 MVI-negative, 19 MVI-positive). These stratifications and their corresponding top-performing features are summarized in Table 4.

### 3.3. Radiomic Feature Analysis

**Figure 2:**
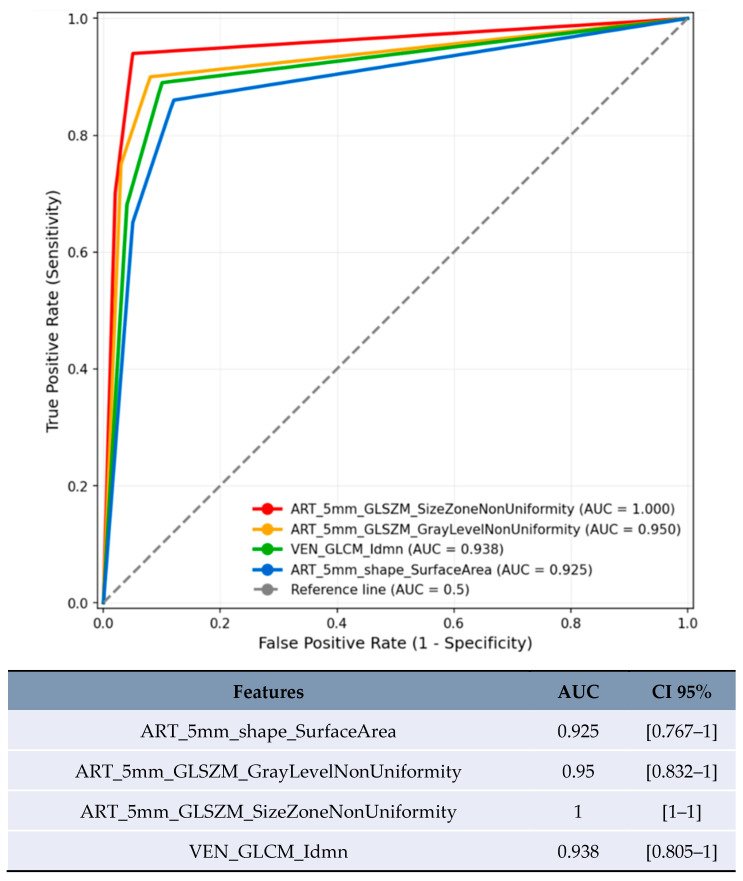
ROC curve of features with the highest AUC in predicting MVI for lesions <20 mm.

**Figure 3:**
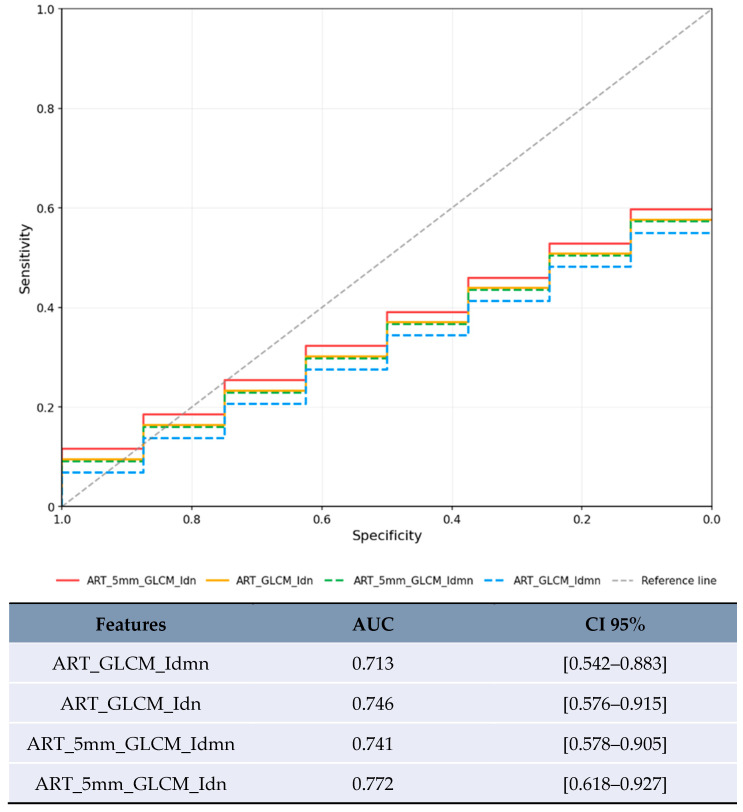
ROC curve with the highest AUCs for the lesion category 20–50 mm.

**Figure 4:**
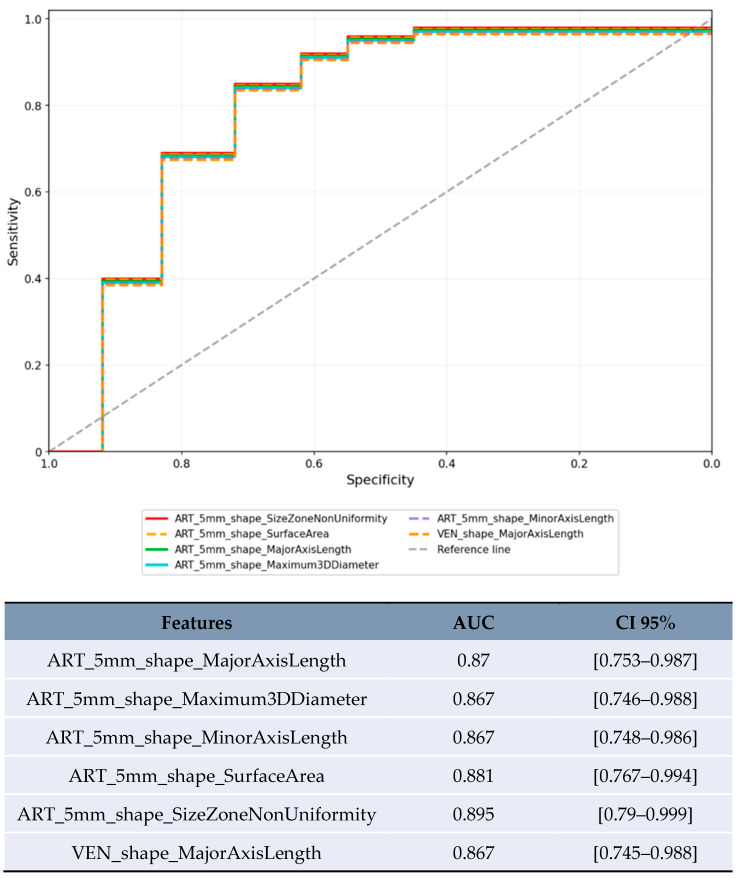
ROC curve for the top-performing features with the highest AUC in the LR-5 category.

Size-based stratification yielded varying numbers of statistically significant features. For lesions <20 mm, 71 features achieved statistical significance (*p* < 0.05), though these results were compromised by severe overfitting as shown in Figure 2. For lesions 20–50 mm, 21 features achieved statistical significance (*p* < 0.05), with the most robust performance (Figure 3). Lesions >50 mm yielded no significant features due to insufficient sample size (*n* = 3). LI-RADS stratification results showed 24 features achieving statistical significance (*p* < 0.05) for LR-3/4 lesions, with delayed phase features demonstrating notable performance. For LR-5 lesions, 182 features achieved statistical significance (*p* < 0.05), with shape-based features showing the highest AUC values (Figure 4).

### 3.4. Top Performing Radiomic Features

For lesions <20 mm in arterial phase with 5 mm margin, several features showed artificially inflated performance metrics that are not clinically meaningful (Figure 2). These results are presented solely for completeness but should not be considered valid findings and are excluded from the conclusions. For lesions 20–50 mm in arterial phase, the most reliable results were obtained with texture homogeneity features (Figure 3): ART_5mm_GLCM_Idn achieved AUC 0.772 [95% CI 0.618–0.927] and ART_5mm_GLCM_Idmn achieved AUC 0.741 [95% CI 0.578–0.905]. The comparison between original and expanded margin features is shown in Table 4. 

**Figure 5:**
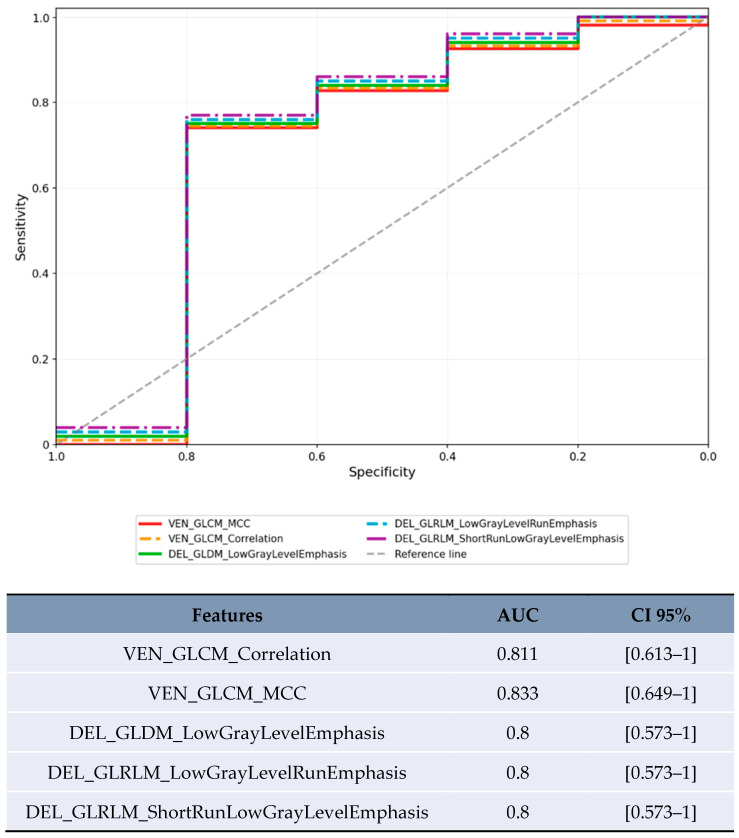
ROC curve for the features with the highest AUC in the LR-3/4 category.

In the limited subset of LR-3/4 lesions (*n* = 20), delayed phase features showed promising preliminary results (Figure 5): GLDM_LowGrayLevelEmphasis achieved AUC 0.8 [95% CI 0.573–1.0] and GLRLM_ShortRunLowGrayLevelEmphasis achieved AUC 0.8 [95% CI 0.573–1.0].

## 4. Discussion

### 4.1. Principal Findings

This study represents a comprehensive investigation of radiomic features across all three phases of contrast-enhanced CT for predicting microvascular invasion in HCC. The identification of delayed phase radiomic features as potential predictors of MVI, shown in Figure 5, represents a preliminary finding requiring validation. The most reliable results were obtained in the 20–50 mm lesion subgroup (Table 4), where arterial phase texture features demonstrated moderate predictive performance.

### 4.2. Clinical Significance of Microvascular Invasion

Microvascular invasion, histologically defined as tumor cell nests within vascular spaces lined by endothelium, represents a key predictor of early HCC recurrence following surgical treatment [16]. In this cohort, 52% of lesions demonstrated MVI (Table 1), consistent with previous reports. Accurate preoperative MVI assessment is essential for optimizing patient prognosis and treatment selection [17]. Current imaging methods have limited accuracy for MVI prediction, making the development of non-invasive predictive tools clinically valuable.

### 4.3. Comparison with Previous Studies

These findings both complement and extend previous radiomic research for MVI prediction, as summarized in Table 5. The results show lower predictive performance compared to larger multicenter studies (Table 5), which likely reflects the smaller sample size and single-center design. Xu et al. [18] achieved superior performance with a cohort ten times larger. The performance is comparable to Zhang et al. [19], though their study benefited from internal validation. The primary distinction of this work is the systematic exploration of delayed phase features (Figure 5), though this represents a preliminary investigation rather than a definitive advancement.

### 4.4. Exploratory Delayed Phase Observations

This study included exploratory analysis of delayed phase imaging, which is routinely acquired in many HCC protocols. In the limited subset of LR-3/4 lesions (*n* = 20), delayed phase features Low Gray Level Emphasis (LGLE) and Short Run Low Gray Level Emphasis (SRLGLE) showed AUC values of 0.8 (Figure 5, Table 4). The biological plausibility of delayed phase features as MVI predictors warrants consideration. Low Gray Level Emphasis in delayed phase may reflect altered contrast washout patterns in areas of microvascular invasion, where disrupted venous drainage and arteriovenous shunting could lead to prolonged contrast retention or altered tissue attenuation. These hemodynamic alterations, while subtle, may be captured by texture analysis even when not visually apparent, as demonstrated in the ROC analysis (Figure 5).

### 4.5. Clinical Implications

Integration of delayed phase radiomics into clinical practice requires careful consideration of risk-benefit ratios. Since delayed phase imaging is already part of standard HCC protocols in many institutions (contributing approximately 2–3 mSv additional radiation dose), utilizing these existing acquisitions for radiomic analysis adds no additional radiation exposure. However, institutions that have eliminated delayed phase imaging to reduce radiation dose should carefully weigh the modest predictive value demonstrated here (AUC 0.8 in limited samples, Table 4) against the additional radiation exposure before reintroducing this phase solely for radiomic analysis. The superior performance of expanded margin features (Table 2) suggests that peritumoral tissue analysis may enhance MVI prediction, consistent with the biological understanding that microvascular invasion often extends beyond visible tumor boundaries. Clinical implementation could follow a standardized workflow: (1) acquisition of standard triphasic CT per institutional protocol; (2) automated segmentation using validated algorithms with radiologist verification; (3) feature extraction using open-source PyRadiomics platform (processing time approximately 5 min per case); (4) application of validated prediction model; (5) integration of MVI risk score into multidisciplinary team discussion. The entire process adds minimal time to routine workflow while providing objective risk stratification. Cost-effectiveness considerations favor radiomic analysis given its use of already-acquired imaging data. No additional scanning, contrast administration, or patient visits are required. The primary investment involves initial software setup and radiologist training, with negligible per-case costs thereafter.

### 4.6. Methodological Considerations

The exploratory analysis of 642 features without correction for multiple testing is a deliberate methodological choice for hypothesis generation. However, readers should interpret all findings with extreme caution. The performance differences between lesion size categories (Table 1) and between original and expanded margins (Table 2) provide insights for future study design. Future validation studies should pre-specify a limited number of candidate features based on these exploratory results and apply appropriate statistical corrections.

### 4.7. Threats to Reproducibility

Several factors in the study design pose significant threats to reproducibility. The high feature-to-sample ratio (12:1) virtually guarantees overfitting, as evidenced by the unreliable results in the <20 mm subgroup (Figure 2). Absence of multiple testing correction means many findings may be false positives. Single-center design with one CT scanner limits technical generalizability. Lack of inter-observer variability assessment for segmentation (acknowledged in Methods Section 2.4). No independent validation cohort. These factors collectively mean that the exact results are unlikely to replicate in independent studies, reinforcing the exploratory nature of this work. The wide confidence intervals observed, particularly in smaller subgroups (Table 4), further emphasize this limitation.

### 4.8. Limitations

The retrospective design and surgical candidate selection may incompletely represent the full spectrum of HCC radiological characteristics. The single-center nature and small sample size (*n* = 49 patients, 54 lesions) limit generalizability. The large number of features analyzed increases the risk of false discoveries. The absence of external validation limits immediate clinical applicability. Additionally, the study population was predominantly male (80%) and had a high prevalence of cirrhosis (71%), which may limit generalizability to other populations.

## 5. Conclusions

This proof-of-concept study demonstrates that radiomic analysis of contrast-enhanced CT provides exploratory insights for predicting microvascular invasion in hepatocellular carcinoma lesions of 20–50 mm size. Novel delayed phase features showed preliminary associations though require extensive validation.

Future validation requires a multicenter prospective study design with: (1) standardized imaging protocols across different scanner manufacturers; (2) pre-specified feature selection based on these exploratory findings; (3) adequate sample size calculation (minimum 10–15 events per feature tested); (4) assessment of inter-observer segmentation variability; (5) external validation cohort; and (6) comparison with existing clinical-radiological prediction models. Only through such rigorous validation can the clinical utility of CT radiomic features for MVI prediction be definitively established.

## Figures and Tables

**Table 1:** Characteristics of All Analyzed Lesions.

Characteristic	Patients (*n* = 54)
Major axis of the lesion, millimeters, median [IQR]	25 [17.75; 40]
Resected segment *	
S2, % (number)	6% (3)
S3, % (number)	9% (5)
S4, % (number)	15% (8)
S5, % (number)	11% (6)
S6, % (number)	21% (11)
S7, % (number)	13% (7)
S8, % (number)	17% (9)
Right hepatic lobe, % (number)	4% (2)
Left hepatic lobe, % (number)	4% (2)
Histological grade	
G1, % (number)	16% (8)
G2, % (number)	66% (33)
G3, % (number)	18% (9)
Capsule, % (number)	30% (16)
Vascular infiltration	
Grade 1, % (number)	39% (21)
Grade 2, % (number)	13% (7)
Clear margin, % (number)	26% (14)
Satellitosis, % (number)	22% (12)

* Note: R0 Resection Margin Indicates Complete Tumor Excision with Microscopically Negative Margins.

**Table 2:** Comparative Performance of Features with and without Margin Expansion (20–50 mm).

Feature	Original Margin AUC	5 mm Expanded Margin AUC	Difference
GLCM_Idn	0.746	0.772	+0.026
GLCM_Idmn	0.713	0.741	+0.028

**Table 3:** Characteristics of Patients with Hepatocellular Carcinoma Included in the Study.

Characteristic	Patients (*n* = 49)
Age at CT, median [IQR]	68 [58.5; 74]
Age at intervention, median [IQR]	68 [59; 74]
Males, % (number)	80% (39)
BCLC Stage	
Stage 0, % (number)	26% (14)
Stage A, % (number)	59% (29)
Stage B, % (number)	13% (6)
Stage C, % (number)	2% (1)
Multiple HCC, % (number)	57% (28)
Cirrhosis, % (number)	71% (35)
Alcohol abuse, % (number)	27% (13)
HCV+, % (number)	45% (22)
HBV+, % (number)	14% (7)
Ascites, % (number)	6% (3)
Encephalopathy, % (number)	2% (1)
sGOT, U/L, median [IQR]	38 [24; 54]
sGPT, U/L, median [IQR]	45 [22.5; 81]
GGT, U/L, median [IQR]	91 [51.25; 165.25]
Albumin, g/dL, median [IQR]	38.9 [33.9; 42.4]
AFP, ng/mL, median [IQR] *	4.5 [0; 7.75]
Total Bilirubin, mg/dL, median [IQR]	0.85 [0.465; 1.285]
INR, median [IQR]	1.11 [1.035; 1.260]
Creatinine, mg/dL, median [IQR]	0.87 [0.715; 1.045]
MELD score, median [IQR]	6 [5; 7]
Lesions per patient	
Patients with 1 lesion, % (number)	90% (44)
Patients with 2 lesions, % (number)	10% (5)

sGOT = serum Glutamic-Oxaloacetic Transaminase, sGPT = serum Glutamic-Pyruvic Transaminase. HBV—Hepatitis B virus; HCV—Hepatitis C virus; INR—International Normalized Ratio; AFP—alpha-fetoprotein; SD—standard deviation; IQR—Interquartile range. * Available for 24 patients.

**Table 4:** Summary of Top-Performing Features by Stratification.

Stratification	Sample Size	MVI+/MVI-	Top Feature	AUC [95% CI]	Interpretation
<20 mm	14	4/10	Not reported	-	Overfitting
20–50 mm	37	21/16	ART_5mm_GLCM_Idn	0.772 [0.618–0.927]	Moderate performance
LR-3/4	20	9/11	DEL_GLDM_LGLE	0.800 [0.573–1.000]	Wide CI, small sample
LR-5	34	19/15	ART_5mm_shape_SZN	0.895 [0.790–0.999]	Requires validation

**Table 5:** Comparison with Previous MVI Prediction Studies.

Study	Sample Size	Features Analyzed	Phases Used	Best AUC	Validation
Xu et al., 2019 [18]	495	1.044	Arterial, Portal	0.909	External
Zhang et al., 2022 [19]	111	Not specified	Multi-phase	0.81	Internal
Renzulli et al., 2016 [20]	125	Qualitative	All-phase	NPV 0.84–0.91	None
Current study	49 (54 lesions)	642	All + Delayed	0.772 (20–50 mm)	None

## Data Availability

The data presented in this study are not publicly available due to patient privacy and ethical restrictions. Access to the data may be granted upon reasonable request to the corresponding author, subject to institutional review board approval and appropriate data sharing agreements.

## References

[1] Sung H., Ferlay J., Siegel R.L., Laversanne M., Soerjomataram I., Jemal A., Bray F. (2021). Global Cancer Statistics 2020, GLOBOCAN Estimates of Incidence and Mortality Worldwide for 36 Cancers in 185 Countries. CA Cancer J. Clin..

[2] Llovet J.M., Kelley R.K., Villanueva A., Singal A.G., Pikarsky E., Roayaie S., Lencioni R., Koike K., Zucman-Rossi J., Finn R.S. (2021). Hepatocellular carcinoma. Nat. Rev. Dis. Primers.

[3] Taddei T.H., Brown D.B., Yarchoan M., Mendiratta-Lala M., Llovet J.M. (2023). AASLD Practice Guidance on prevention, diagnosis, and treatment of hepatocellular carcinoma. Hepatology.

[4] Ruf A., Dirchwolf M., Freeman R. (2022). From Child-Pugh to MELD Score and Beyond: Taking a Walk Down Memory Lane. Ann. Hepatol..

[5] European Association for the Study of the Liver (2018). EASL Clinical Practice Guidelines: Management of hepatocellular carcinoma. J. Hepatol..

[6] Reig M., Forner A., Rimola J., Ferrer-Fàbrega J., Burrel M., Garcia-Criado Á., Kelley R.K., Galle P.R., Mazzaferro V., Salem R. (2022). BCLC strategy for prognosis prediction and treatment recommendation: The 2022 update. J. Hepatol..

[7] Cabibbo G., Daniele B., Borzio M., Casadei-Gardini A., Cillo U., Colli A., Conforti M., Dadduzio V., Dionisi F., Farinati F. (2024). Multidisciplinary Treatment of Hepatocellular Carcinoma in 2023, Italian practice Treatment Guidelines. Dig. Liver Dis..

[8] Wei J., Jiang H., Gu D., Niu M., Fu F., Han Y., Song B., Tian J. (2020). Radiomics in liver diseases: Current progress and future opportunities. Liver Int..

[9] Wang W., Chen Q., Iwamoto Y., Han X., Zhang Q., Hu H., Lin L., Chen Y.-W. (2019). Deep Learning-Based Radiomics Models for Early Recurrence Prediction of Hepatocellular Carcinoma with Multi-phase CT Images and Clinical Data. Annu. Int. Conf. IEEE Eng. Med. Biol. Soc..

[10] Rodriguez-Peralvarez M., Luong T.V., Andreana L., Meyer T., Dhillon A.P., Burroughs A.K. (2013). A systematic review of microvascular invasion in hepatocellular carcinoma: Diagnostic and prognostic variability. Ann. Surg. Oncol..

[11] Lei Z., Li J., Wu D., Xia Y., Wang Q., Si A., Wang K., Wan X., Lau W.Y., Wu M. (2016). Nomogram for preoperative estimation of microvascular invasion risk in hepatitis B virus-related hepatocellular carcinoma within the Milan criteria. JAMA Surg..

[12] Yang L., Gu D., Wei J., Yang C., Rao S., Wang W., Chen C., Ding Y., Tian J., Zeng M. (2019). A Radiomics Nomogram for Preoperative Prediction of Microvascular Invasion in Hepatocellular Carcinoma. Liver Cancer.

[13] Gillies R.J., Kinahan P.E., Hricak H. (2016). Radiomics: Images are more than pictures, they are data. Radiology.

[14] Brancato V., Cerrone M., Garbino N., Salvatore M., Cavaliere C. (2024). Current status of magnetic resonance imaging radiomics in hepatocellular carcinoma: A quantitative review with Radiomics Quality Score. World J. Gastroenterol..

[15] Cunha G.M., Sirlin C.B., Fowler K.J. (2021). Imaging diagnosis of hepatocellular carcinoma: LI-RADS. Chin. Clin. Oncol..

[16] Garzali I.U., I Carr B., İnCe V., Işık B., Akatlı A.N., Yılmaz S. (2024). Microvascular invasion in hepatocellular carcinoma: Some puzzling facets. Turk. J. Gastroenterol..

[17] Tong X., Li J. (2022). Noninvasively predict the micro-vascular invasion and histopathological grade of hepatocellular carcinoma with CT-derived radiomics. Eur. J. Radiol. Open..

[18] Xu X., Zhang H.-L., Liu Q.-P., Sun S.-W., Zhang J., Zhu F.-P., Yang G., Yan X., Zhang Y.-D., Liu X.-S. (2019). Radiomic analysis of contrast-enhanced CT predicts microvascular invasion and outcome in hepatocellular carcinoma. J. Hepatol..

[19] Zhang S., Duan C., Zhou X., Liu F., Wang X., Shao Q., Gao Y., Duan F., Zhao R., Wang G. (2022). Radiomics nomogram for prediction of microvascular invasion in hepatocellular carcinoma based on MR imaging with Gd-EOB-DTPA. Front. Oncol..

[20] Renzulli M., Brocchi S., Cucchetti A., Mazzotti F., Mosconi C., Sportoletti C., Brandi G., Pinna A.D., Golfieri R. (2016). Can Current Preoperative Imaging Be Used to Detect Microvascular Invasion of Hepatocellular Carcinoma?. Radiology.

